# Awareness of the relationship between microbiota and exercise: an education-based evaluation

**DOI:** 10.1590/1806-9282.20260054

**Published:** 2026-06-22

**Authors:** Ozgenur Hacioglu, Burcu Ozuberk, Serhan Sakarya

**Affiliations:** 1 Kirklareli University, Faculty of Health Sciences – Kırklareli, Türkiye.; 2 Kirklareli University, Faculty of Health Sciences, Department of Physical Therapy and Rehabilitation – Kırklareli, Türkiye.; 3 Medicana International Hospital – İzmir, Türkiye.

**Keywords:** Microbiota, Exercise, Health education, Awareness, Educational program

## Abstract

**OBJECTIVE:**

Individuals who are cognizant of microbiota and exercise connections may come to understand that exercise not only benefits their physical health but also has favorable effects on their internal balance and microbiota. In this context, the aim of this educational study was to illustrate how the balance between exercise and microbiota functions for the promotion of a healthy life.

**METHODS:**

The training sessions were conducted for 2 h per week and took place face-to-face over a period of 4 weeks. The data collection instruments used in the study included the Microbiota Awareness Scale, the Exercise Benefits/Barriers Scale, and the International Physical Activity Questionnaire-Short Form to assess participants’ current levels of physical activity. All scales, except for the International Physical Activity Questionnaire-Short Form, were administered to the participants during both the pre-test and post-test. Statistical Package for the Social Sciences 25.0 was used for data analysis.

**RESULTS:**

A total of 47 students participated in the education. The average age of the participants was 23±1.6 years. The students were found to be very active, with a mean physical activity level score of 8,685.36±6,684.09 Metabolic Equivalent-min/week. The average score of students in the microbiota knowledge pre-test was 42.5±1.4 out of 100, while the average score in the post-test was determined to be 87.5±3.2. The average score of the Exercise Benefit/Barrier Scale before the training was 86±24.97, while after the training it was 90.18±22.10.

**CONCLUSION:**

It has been determined that the applied microbiota and exercise training helped participants establish a balance between exercise and microbiota for a healthy life.

## INTRODUCTION

The microbiota refers to the community of all microorganisms living in our body. The human microbiota consists of bacteria, viruses, fungi, and other microorganisms found primarily in the intestines, as well as on the skin, in the mouth, respiratory tract, and other mucosal surfaces^1^. It is known that the number of microorganisms in the human body exceeds the number of human cells. The microbiota is an ecosystem that plays a central role in human health. It has a significant impact on digestion, immunity, metabolism, and even brain functions^2,3^.

Various studies have reported that daily choices related to diet, exercise, and hygiene can affect the human microbiota^4–7^. Exercise is one of the many factors that improve human health. It is known that exercise strengthens the immune system, supports heart health, and has positive effects on overall health. Many studies have shown that regular exercise can increase the diversity of the gut microbiota^8–10^. Microbiota diversity is crucial for overall health because a more diverse microbiota is known to increase the body's ability to fight different diseases. Studies have shown that regular exercise can help beneficial bacteria proliferate, which may improve immune system functions and digestive health^6,8,10^.

Recent studies on the relationship between microbiota and human health have contributed to a better understanding of how these microorganisms affect an individual's overall health^1–3^. Therefore, research on microbiota and exercise provides an important foundation for individuals to adopt healthy lifestyles. Microbiota and exercise awareness can help individuals better understand the microorganisms in their bodies and encourage them to develop healthier lifestyle habits through exercise^8–10^. For example, individuals with digestive issues can be educated to avoid habits that could disrupt microbiota balance, and they can be encouraged to exercise^11^.

Education can help individuals adopt microbiota-friendly approaches, such as probiotics, prebiotics, and a balanced diet. Additionally, individuals who are informed about the effects of stress on the microbiota may turn to stress management methods, leading to positive effects on their digestive and overall health^11–13^. Microbiota education not only impacts individual health but also raises health awareness at the societal level. This awareness, created among individuals from different professions and age groups, can have a significant impact, especially in terms of promoting healthy lifestyles and disease prevention^12^. The aim of this study is to comprehensively examine the relationship between microbiota, exercise, and health among university students, and to enhance their knowledge of microbiota and increase awareness of the benefits of exercise.

## METHODS

### Study design and participant recruitment

This study was designed as a non-clinical, education-based study aimed at increasing awareness of the relationship between exercise and microbiota. The research was conducted in a quasi-experimental design with 47 students from the faculty of engineering through face-to-face sessions. The training sessions were held twice a week for 2 h each and took place over a period of four weeks. The inclusion criteria for the study were engineering department university students who had no communication issues, no chronic cardiac or pulmonary diseases, the ability to attend the training sessions regularly, and who had signed the voluntary consent form.

### Data collection tools

Participants were provided with a microbiota and exercise training plan, as well as a personal information form, and were asked to fill them out. The data collection tools used in the study were the Microbiota Awareness Scale^14^, the Exercise Benefit/Barrier Scale^15^, and the International Physical Activity Questionnaire-Short Form^16^ to determine the students' current physical activity levels. The Metabolic Equivalent (MET) method was used to determine the physical activity level. The scales used in the study were administered to students in both the pre-test and post-test. Only the International Physical Activity Questionnaire-Short Form was administered once in the pre-test to determine the students' current physical activity levels. All scales were applied to the participants 3 months after the training and compared with the scores before and after the training.

### Evaluation of data

The data obtained from the research were analyzed using the Statistical Package for the Social Sciences 25 (SPSS 25.0) program. Descriptive statistical methods (frequency, percentage, minimum-maximum values, median, mean, and standard deviation) were utilized in evaluating the data, and the normal distribution of the data was assessed using the Kolmogorov-Smirnov test. The independent samples t-test was used for data showing normal distribution. Results were evaluated at a significance level of p<0.05 and p<0.001 with a 95%CI.

### Ethical aspect

Ethics committee approval to conduct the present study was obtained from the Kirklareli University Scientific Research and Publication Ethics Committee (Number: E-35523585-199-113120, dated February 08, 2024). The purpose and method of the study were explained to the participants before its commencement. All participants gave written informed consent before inclusion. All practices were carried out in accordance with the 1964 Helsinki Declaration.

## RESULTS

### Evaluation of participant information form

The sociodemographic characteristics of the participants are shown in Table 1, and a total of 47 engineering students participated in the training. The mean age of the participants was 23±1.6 years, with the oldest being 27 and the youngest 21. When the sociodemographic characteristics of the participants were examined, it was found that 40 individuals did not regularly consume probiotic products, 31 individuals believed they did not have regular and adequate eating habits, and 24 individuals did not engage in regular exercise. It was determined that none of the students had a diagnosed chronic illness. It was observed that the number of students who thought their intestinal health was generally good (11/47) was low. 30 students were non-smokers, while 17 students were smokers. 41 students had never heard of the concept of microbiota, and all of the students had not participated in any training on microbiota, exercise, and healthy living. The students were found to be very active, with a mean physical activity level score of 8,685.36±6,684.09 MET-min/week.

**Table 1:** Socio-demographic characteristics of the participants.

	Variables	n
Age (mean: 23±1.6 [SD])	Min.	21
	Max.	27
Gender	Woman	4
	Man	43
	Total	47
Height (mean:1.76±8.9 [SD])	Min.	1.53
	Max.	1.95
Weight (mean:74.6±15.6)	Min.	42
	Max.	115
Educational status	Bachelors degree	47
Do you regularly consume probiotic products?	Yes	7
	No	40
Do you think that you have regular and adequate eating habits?	Yes	16
	No	31
Do you smoke?	Yes	17
	No	30
Do you exercise regularly?	Yes	23
	No	24
Do you have any chronic diseases?	No	47
How would you generally assess your intestinal health?	Bad	36
	Good	11
Have you heard of the concept of microbiota before?	Yes	6
	No	41
Have you ever attended a training related to microbiota, exercise, and healthy living?	No	47
IPAQ (MET-min/week)	Min.	1,890
	Max.	25,074
	Mean±SD	8,685.36 ± 6,684.09

Data are represented as mean±SD. Mean: arithmetic mean; SD: standard deviation; Min: minimum; Max: maximum; IPAQ: Internatıonal Physical Activity Questionnaire; MET: Metabolic Equivalent.

### Pre-test application

It was determined that the average score of the students in the microbiota knowledge test pre-test application was 42.5±1.4, above 100. The average score of the students in the exercise benefit/barrier scale pre-test application was 86±24.97 (Table 2).

**Table 2:** Comparison of participants' Microbiota Awareness Scale and Exercise Benefit/Barrier Scale scores before and after training.

Variables	Pre-test (mean±SD)	Post-test (mean±SD)	p*
MAS	42.5±1.4	87.5±3.2	<0.001*
EBBS	86±24.97	90.18±22.10	>0.001
EBenS	57.63±24.30	58.18±25.11	>0.001
EBarS	27.81±7.13	32.54±11.07	>0.001

Data are represented as Mean±SD. Mean: arithmetic mean; SD: standard deviation; MAS: Microbiota Awareness Scale; EBBS: Exercise Benefit/Barrier Scale; EBenS: Exercise Benefit Scale; EBarS: Exercise Barrier Scale.

* Independent samples t-test.

### Post-test application

In the post-test application of the microbiota knowledge test, the average score obtained by participants was 87.5±3.2, above 100. The average score of the students in the exercise benefit/barrier scale post-test application was 86±24.97 (Table 2).

The distribution of total score and sub-dimension score averages of the students' microbiota awareness scale is shown in Table 3. The total score of the Microbiota Awareness Scale was 23.1±5.1, with sub-dimensions as follows: General Knowledge 28.8±0.8; Product Knowledge 16.5±1.4; Chronic Disease 23.3±1.0; Probiotic and Prebiotic Nomenclature 23.9±0.8. The distribution of total score and sub-dimension score averages of the students' exercise benefit/barrier scale is shown in Table 3. The total score of the Exercise Benefit/Barrier Scale scale was 88.09±23.40, with sub-dimensions as follows: Exercise Benefit Scale 57.90±24.42; Exercise Barrier Scale 30.18±9.5.

**Table 3:** Distribution of total and subdimensions score means of the Microbiota Awareness Scale and the Exercise Benefit/Barrier Scale of participants (n=47).

MAS and subdimensions	Mean±SD	Min	Max
General information	28.8±0.8	27	30
Product information	16.5±1.4	13	19
Chronic disease	23.3±1.0	22	25
Probiotic and prebiotic nomenclature	23.9±0.8	23	25
MAS total score	23.1±5.1	16.5	28.8
EBBS and subdimensions	Mean±SD	Min	Max
EBenS	57.90±24.42	29	116
EBenS	57.90±24.42	29	116
EBarS	30.18±9.51	14	56
EBBS	88.09±23.40	49	131

Data are represented as mean±SD. Mean: arithmetic mean; SD: standard deviation; Min: minimum; Max: maximum; MAS: Microbiota Awareness Scale; EBBS: Exercise Benefit/Barrier Scale; EBenS: Exercise Benefit Scale; EBarS: Exercise Barrier Scale.

A statistically significant difference was found in the microbiota awareness scale before and after the training, with the participants' post-training mean scores being higher (p<0.001). However, there was not a statistically significant difference in the exercise benefit/barrier scale (p>0.001).

## DISCUSSION

Microbiota varies from individual to individual, and it is known that genetic factors, diet, antibiotic use, lifestyle, and environmental factors influence this diversity^1,2^. Recent studies have shown that regular exercise can increase the diversity of beneficial microorganisms in the gut microbiota and help maintain the balance of the microbiota, thereby strengthening the immune system. Furthermore, regular exercise has been reported to contribute to the prevention of obesity and other metabolic diseases by altering the role of the microbiota in fat storage and hunger sensation^5–8^.

It is known that individuals' knowledge levels about microbiota, which is of critical importance to health, can vary based on factors such as education level, age, and profession. Having basic knowledge about microbiota enables individuals to make more informed decisions regarding their bodies and overall health. For instance, habits such as diet choices and antibiotic use can directly affect the microbiota^2,5,11–13^. Various studies in the literature suggest that awareness of microbiota, probiotics, and prebiotics may be higher among students in health-related fields^11–13^. In this study, the average score of the microbiota awareness scale applied to students before the training was found to be 42.5/100. After the training, the score increased to 87.5/100, indicating that the students' knowledge had improved. The low pre-training knowledge scores of the students may be attributed to their fields of study being non-health-related. After the training, it was determined that students had learned basic concepts such as microbiota and probiotics, were able to establish the microbiota-health relationship, and had incorporated probiotics into their diets.

In our study, although there was no statistically significant difference in the students' general exercise perception levels, an increase in value was observed. This change can be understood from the increase in the belief in the benefits of exercise and the decrease in the perception of barriers to exercise, which are sub-parameters of the scale. In the literature, the results of exercise perception among health professionals are similar to the results of engineering faculty students in our study^17^. In previous studies, it is not surprising that physical activity levels in physiotherapy students are above the general average, whereas it is surprising that engineering faculty students also exhibit high levels of physical activity^17–19^. This is because physical activity and exercise occupy a much more important and effective place in physiotherapy and rehabilitation education, and therefore, it is expected that physiotherapy students have higher physical activity levels than the general average. In our study, the high levels of physical activity in engineering faculty students can be attributed to field trips, workshop activities, and projects within the campus that require active movement, which engineers typically engage in. The development of exercise routines among them may also reflect the need for stress relief through physical activity due to their competitive nature.

The relationship between exercise and microbiota was understood by the students both theoretically and practically. Providing microbiota education to individuals in non-health-related professions can help them gain awareness in this area and adopt healthy habits in their daily lives. This awareness is an important step in increasing health consciousness.

## CONCLUSION

Providing individuals with awareness of microbiota and exercise through an educational program can not only improve individual health and quality of life but also contribute to the increase of public health consciousness. It is believed that this education can assist individuals in making more informed decisions regarding their bodies, while also contributing to the spread of healthy lifestyles at the societal level.

## Data Availability

The datasets generated and/or analyzed during the current study are available from the corresponding author upon reasonable request.
